# Elevated triglyceride-glucose index associated with increased risk of diabetes in non-obese young adults: a longitudinal retrospective cohort study from multiple Asian countries

**DOI:** 10.3389/fendo.2024.1427207

**Published:** 2024-08-08

**Authors:** Jian Han, Weifeng Dai, Lixia Chen, Zhenhua Huang, Chengzhi Li, Keke Wang

**Affiliations:** ^1^ Department of Interventional Radiology and Vascular Surgery, The First Affiliated Hospital of Jinan University, Guangzhou, Guangdong, China; ^2^ Department of Cardiology, The First Affiliated Hospital of Jinan University, Guangzhou, Guangdong, China; ^3^ Department of Emergency Medicine, The First Affiliated Hospital of Shenzhen University, Shenzhen Second People’s Hospital, Shenzhen, China; ^4^ Department of Emergency Medicine, The First Affiliated Hospital, Sun Yet-sen University, Guangzhou, China

**Keywords:** TyG index, association, diabetes, non-obese young adults, non-linear

## Abstract

**Objective:**

Previous studies have confirmed a positive correlation between the Triglyceride-Glucose (TyG) index and future risk of diabetes. However, evidence of this association in non-obese young populations remains limited. This study aims to investigate the relationship between the TyG index and the future risk of diabetes among non-obese young adults.

**Methods:**

This retrospective cohort study included 113,509 non-obese young adults from China and 9,549 from Japan. The mean age was 35.73 ± 6.38 years, and 56,469 participants (45.89%) were male. The median follow-up duration was 3.38 years. The association between baseline TyG index and risk of diabetes was examined using Cox proportional hazards regression models. Non-linear relationships between the TyG index and risk of diabetes were identified using cubic splines and smoothed curve fitting in the Cox models. Sensitivity and subgroup analyses were also conducted.

**Results:**

After adjusting for covariates, the results indicated a positive correlation between the TyG index and risk of diabetes in non-obese young adults (HR=3.57, 95% CI: 2.92-4.36, P<0.0001). A non-linear relationship was observed with an inflection point at 7.3. The HR to the right of this inflection point was 3.70 (95% CI: 3.02-4.52, P<0.0001), while to the left, it was 0.34 (95% CI: 0.06-1.88, P=0.2161). The robustness of our findings was confirmed through a series of sensitivity analyses and subgroup analyses.

**Conclusion:**

This study reveals a positive and non-linear association between the TyG index and risk of diabetes among non-obese young adults. Interventions aimed at reducing the TyG index by lowering triglycerides or fasting glucose levels could substantially decrease the future likelihood of developing diabetes in this population.

## Introduction

The Triglyceride-Glucose (TyG) index, a marker used to evaluate insulin resistance, combines levels of plasma triglycerides with fasting glucose (1). Compared to other insulin resistance markers, the TyG index is simple, cost-effective, and has been shown through numerous studies to be an important predictor of Type 2 Diabetes Mellitus (T2DM) in various populations due to its high sensitivity and specificity (2–6). Additionally, the TyG index has been linked to increased risks of metabolic syndrome, atherosclerosis, and coronary heart disease (7–9).

Diabetes is a chronic disease characterized by persistently elevated levels of blood glucose. It is classified into Type 2 and Type 1 diabetes, with the latter being the predominant form. As of 2021, the International Diabetes Federation reported that around 537 million adults globally had diabetes, with estimates forecasting a jump to 783 million by 2045 (10). In Asia, especially in China, the prevalence of diabetes is notably high; data from 2020 show an adult prevalence of approximately 12.8%, translating to about 116 million people (11). Diabetes not only causes severe individual health problems such as retinopathy, nephropathy, and cardiovascular diseases (12–14), but also imposes a significant socio-economic burden (15).

Despite having a normal weight, non-obese young adults are not entirely immune to the risk of diabetes. Recently, with lifestyle changes, the incidence of diabetes among this group has increased. Studies suggest that unlike the prevalence of obesity-related diabetes in Western countries, the incidence of diabetes in non-obese young Asians is on the rise (6, 16). This phenomenon may be related to genetic factors, dietary habits, and decreased physical activity. Particularly in urban areas, fast-paced lifestyles, and diets high in carbohydrates and sugars have exacerbated this trend.

Numerous studies have indicated an association between the TyG index and risk of diabetes (17–22). However, these studies have primarily focused on the general or obese populations, with fewer studies concentrating on non-obese young adults. Although there is evidence of the TyG index’s correlation with risk of diabetes, its specific role in non-obese young adults remains unclear. Thus, further research into the association between the TyG index and risk of diabetes in this population is necessary.

Therefore, given the large populations in Asian countries, particularly China and Japan, this study aims to explore the association between the TyG index and risk of diabetes among non-obese young adults. By conducting this retrospective cohort study, we can better understand the risk factors for diabetes in this population and develop appropriate preventive strategies.

## Methods

### Study design

This study employed a retrospective cohort design, utilizing data from a Chinese computer database by researchers Chen et al. (23), and the NAGALA (Non-Alcoholic Fatty Liver Disease in Gifu Area, Longitudinal Analysis) database established at the Murakami Memorial Hospital in Japan (24). The primary independent variable was the baseline TyG index. The outcome variable was diabetes, recorded as a binary variable (0 = normal, 1 = diabetes).

### Data source

The original data for this study were sourced from the DATADRYAD database (www.datadryad.org). The data concerning Chinese individuals came from a

published article entitled “Association of body mass index and age with incident diabetes in Chinese adults: a population-based cohort study” referred to as the Dryad dataset (https://doi.org/10.5061/dryad.ft8750v) (23). Data on Japanese individuals were derived from a study on adults in Japan, article entitled “Ectopic fat obesity presents the greatest risk for incident type 2 diabetes: a population-based longitudinal study” (24), Dryad dataset (https://doi.org/10.5061/dryad.8q0p192). Dryad’s terms of service permit the secondary analysis of data by other researchers without infringing upon the authors’ rights.

### Study population

**Figure 1:**
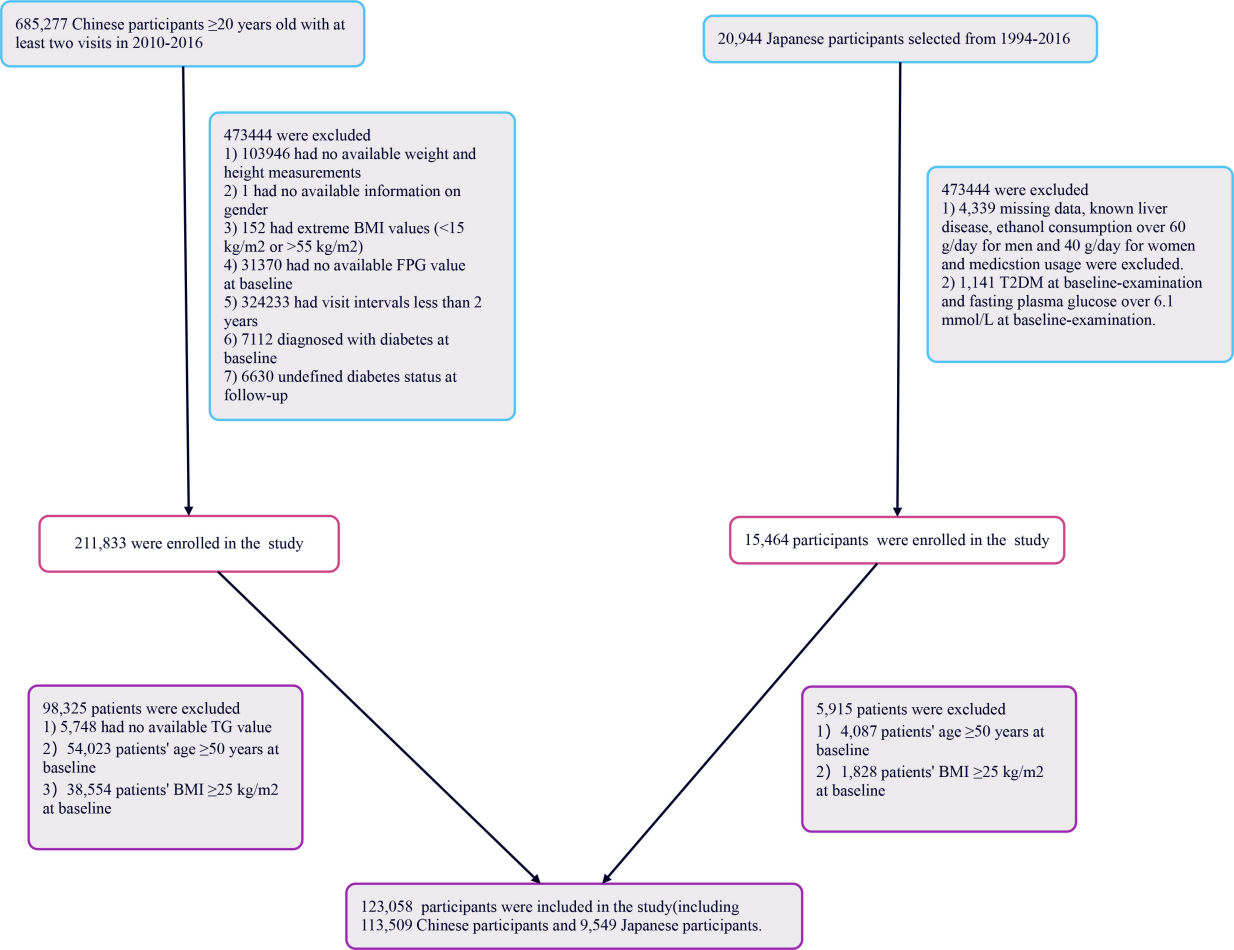
Flowchart of study participants.

**Figure 1** illustrates the initial inclusion of 685,277 participants in the Chinese cohort, with 473,744 excluded from the primary study, leaving 211,833 for analysis. The Japanese cohort began with 20,944 participants; 5,480 were excluded, resulting in 15,464 individuals analyzed. Ultimately, 227,297 participants were included in the study. The exclusion criteria for the current analysis were as follows: (i) participants lacking triglyceride data were excluded (n=5,748); (ii) individuals aged 50 years or older were omitted (n=58,110); (iii) those with a body mass index (BMI) of 25 kg/m^2^ or higher were excluded to focus on a non-obese population (n=40,382). The final participant count was 123,058, comprising 113,509 from China and 9,549 from Japan. This research respected the principles outlined in the Declaration of Helsinki, with all procedures aligning with the relevant protocols and rules as specified in the declaration segment. As a result of its retrospective design, ethical consent or informed approval was not necessary from the institutional review board for the analysis of this secondary dataset.

### Data collection

In this study, we collected data from both Chinese and Japanese populations, focusing on shared variables such as demographic characteristics (age and gender), fasting plasma glucose (FBG), BMI, alanine aminotransferase (ALT), systolic blood pressures (SBP), triglycerides (TG), total cholesterol (TC), diastolic blood pressures (DBP), high-density lipoprotein cholesterol (HDL-c), and aspartate aminotransferase (AST), as well as follow-up duration. Fasting venous blood samples were collected after a minimum 10-hour fast at each visit. Blood pressure was measured using a standard mercury sphygmomanometer. BMI was calculated as weight in kilograms divided by the square of height in meters. Covariates were selected based on clinical experience and published literature, leading us to include the following variables: continuous variables such as DBP, FBG, BMI, AST, HDL-c, SBP, TG, TC, and ALT; and categorical variables such as gender.

### Definitions

BMI (kg/m 2) = body weight (kg)/height ^2^ (m ^2^). Non-obesity is defined as BMI <25 kg/m2 (24, 25). Young adults are defined as individuals aged 20-49 years (26). The TyG index was treated as a continuous variable and calculated using the formula: TyG index = ln [(fasting serum triglycerides in mg/dL) × (fasting plasma glucose in mg/dL)/2] (27, 28).

### Outcome measures

The primary outcome was determined during the follow-up period and was whether participants were diagnosed with diabetes, which was recorded as a binary variable (0 = no diabetes, 1 = diabetes).

### Statistical analysis

The study included participants who were classified into four groups based on their TyG index values. The averages ± deviations (for normally distributed data) or medians (with interquartile ranges) (for skewed data) were reported for continuous variables. Categorical data was presented as frequencies and percentages. The analysis involved using the χ2 test for categorical variables, and either one-way ANOVA (for normal data) or the Kruskal-Wallis H test (for skewed data) to compare differences between the TyG index groups. Survival rates and time-to-event variables were determined through the Kaplan-Meier method, and the log-rank test was employed to compare diabetes-free survival among the TyG index groups.

To investigate the association between the TyG index and the risk of diabetes, we executed both univariate and multivariate Cox proportional hazards models, adjusting for confounders as identified through clinical insights, literature review, analyses of individual variables, and checks for multicollinearity. We undertook a variety of sensitivity analyses to assure the stability of our findings. Initially, we removed participants with an SBP over 140 mmHg. Further sensitivity analyses were done after excluding individuals with a BBP of 140 mmHg or higher. We also investigated the TyG index’s relationship with risk of diabetes without including women in the adjusted covariates. Additionally, to secure the robustness of our findings, we utilized Generalized Additive Models (GAM) to integrate continuous variables into the models as curves.

Furthermore, we applied Cox regression models equipped with cubic splines to better understand the non-linear connections between the TyG index and diabetes. A piecewise Cox regression approach was also employed to elaborate on these non-linear associations. The optimal model to describe the relation between the TyG index and risk of diabetes was pinpointed using the log-likelihood ratio test. For detailed examination, stratified Cox models were used in subgroup analysis, and the existence of interaction terms was confirmed via the likelihood ratio test.

The R software package (http://www.r-project.org, R Foundation) and Empower Stats (X&Y Solutions, Inc., Boston, MA, http://www.empowerstats.com) were utilized for the conducted analyses. Statistical significance was determined with a P-value below 0.05.

## Result

### Characteristics of participants

**Figure 2:**
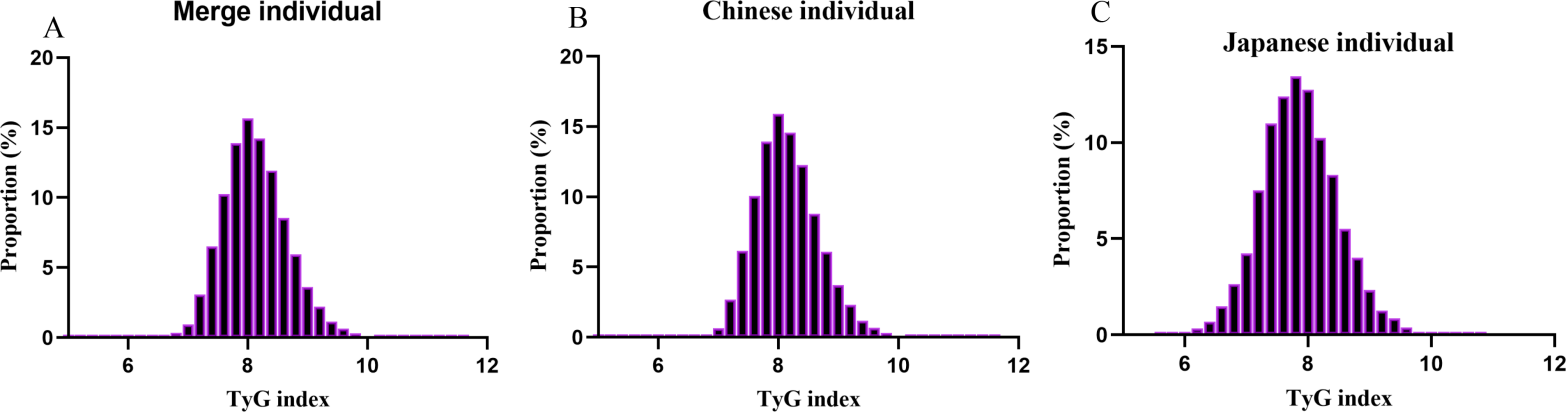
Distribution of TyG index. **(A)** showed that TyG index for merge individuals presented a normal distribution ranging from 4.67 to 11.60, with a mean level of 8.13. **(B)** indicated that TyG index for Chinese presented a normal distribution ranging from 4.67 to 11.6, with a mean level of 8.15. **(C)** indicated that TyG index for Japanese presented a normal distribution ranging from 5.63 to 10.73, with a median level of 7.86.

**Table 1** displays the demographic and clinical characteristics of the individuals involved in the study, along with **Supplementary Tables 1**, **2**. The average age recorded was 35.73 ± 6.38 years, with 56,469 participants (45.89%) identified as male. The median follow-up period amounted to 3.38 years, during which 515 individuals (0.42%) were diagnosed with diabetes. The TyG index values were distributed between 4.67 to 11.60, with an average level of 8.13 (**Figure 2A**). As the TyG index quartile increased, significant increments were observed in DBP, age, FBG, ALT, BMI, TG, SBP, TC, and AST, correlating with a decline in HDL-c levels (all p-values < 0.001). The prevalence of diabetes mellitus also rose considerably from Q1 to Q4 (0.24% to 0.91%, p < 0.001). Notably, there was a notable gender variation, with higher proportions of males in the upper TyG index quartiles (**Table 1**). In the case of Japanese participants, comparable patterns emerged, showcasing substantial rises in metabolic risk factors and diabetes incidence as the TyG index quartile progressed from Q1 to Q4. The TyG index spanned from 5.63 to 10.73, with a median value of 7.86 (**Figure 2C**). The duration of follow-up was extended among Japanese participants, indicating a prolonged period for potential diabetes development (**Supplementary Table 1**). Likewise, Chinese participants exhibited trends mirroring those of the total population, experiencing significant elevations in metabolic risk factors and diabetes incidence rates across TyG index quartiles (**Supplementary Table 2**). The TyG index was dispersed from 4.67 to 11.6, with an average level of 8.15 (**Figure 2B**).

**Table 1:** The baseline characteristics of participants.

TyG index (quartile)	Q1 (≤7.94)	Q2 (7.95-8.33)	Q3 (8.38-8.77)	Q4 (≥8.78)	P-value
**participants**	30,728	30,796	30,767	30,767	
**Age (years)**	34.90 ± 6.20	35.20 ± 6.33	35.70 ± 6.38	37.13 ± 6.36	<0.001
**BMI (kg/m2)**	20.51 ± 1.97	20.95 ± 2.04	21.47 ± 2.03	22.45 ± 1.80	<0.001
**SBP (mmHg)**	109.71 ± 12.65	111.80 ± 13.00	113.91 ± 13.24	117.74 ± 13.65	<0.001
**DBP (mmHg)**	68.37 ± 8.93	69.72 ± 9.09	71.17 ± 9.20	73.97 ± 9.74	<0.001
**FBG (mg/dL)**	83.18 ± 9.27	85.44 ± 9.31	87.78 ± 9.00	90.75 ± 9.64	<0.001
**TyG index**	7.48 ± 0.23	7.93 ± 0.09	8.26 ± 0.11	8.85 ± 0.35	<0.001
**TG (mg/dL)**	44.06 ± 9.88	65.85 ± 9.52	89.88 ± 13.20	165.63 ± 83.73	<0.001
**ALT (U/L)**	19.00 (16.00-22.00)	19.00 (16.60-22.80)	20.00 (17.00-24.00)	22.00 (18.40-26.70)	<0.001
**AST (U/L)**	13.00 (10.30-17.00)	14.00 (10.95-19.00)	15.70 (11.80-22.10)	20.80 (14.50-30.60)	<0.001
**TC (mg/dL)**	164.24 ± 28.41	170.36 ± 29.40	176.28 ± 30.51	188.84 ± 33.66	<0.001
**HDL-c (mg/dL)**	46.40 (0.00-60.71)	42.54 (0.00-57.23)	41.38 (0.00-54.91)	37.90 (0.00-50.66)	<0.001
Gender					<0.001
**Male**	8,044 (26.18%)	11,481 (37.28%)	15,394 (50.03%)	21,550 (70.04%)	
**Female**	22,684 (73.82%)	19,315 (62.72%)	15,373 (49.97%)	9,217 (29.96%)	
**Follow-up (year)**	3.63 ± 1.88	3.36 ± 1.59	3.28 ± 1.54	3.23 ± 1.50	<0.001
**Incident of diabetes**	49 (0.16%)	55 (0.18%)	78 (0.25%)	333 (1.08%)	<0.001

Continuous variables were summarized as mean (SD) or medians (quartile interval); categorical variables were displayed as percentage (%).

BMI, body mass index; SBP, systolic blood pressure; DBP; diastolic blood pressure; TG triglyceride; AST aspartate aminotransferase; ALT, alanine aminotransferase; BUN, blood urea nitrogen; Scr, serum creatinine; FBG, fasting plasma glucose; TyG index, triglyceride glucose index.

### The relationship between TyG index quartiles and diabetes incidence

**Figure 3:**
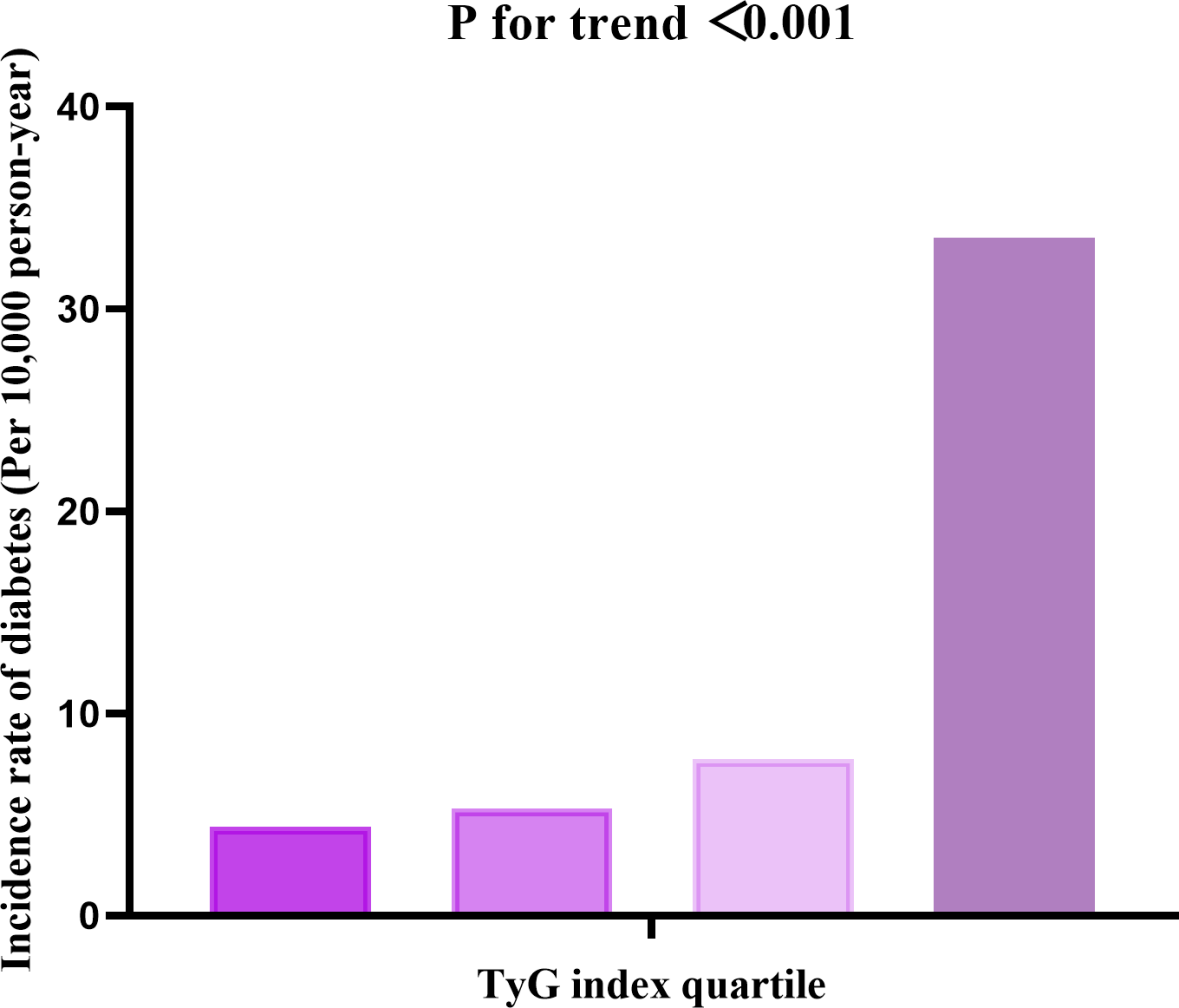
The incidence rate for diabetes (Per 10,000 person-year) according to the quartiles of TyG index. Participants with the highest TyG index (Q4) had higher diabetes incidence rates than those with the lowest TyG index (Q1) (P < 0.001 for trend).

**Figure 4:**
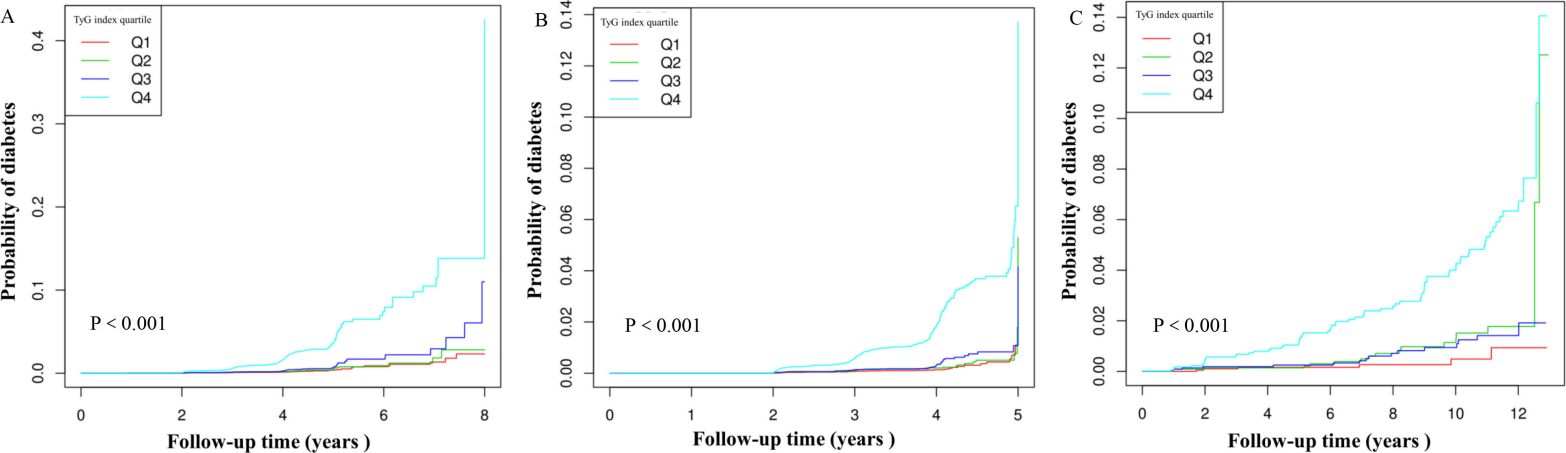
Kaplan–Meier curves for the probability of diabetes. The **A** for Chinese and Japanese individuals; The **B** for Chinese individuals; The **C** for Japanese individuals. The probability of diabetes increased progressively with rising TyG index, meaning that Patients with the highest TyG index had the higher probability of diabetes in non-obese young individual.

Based on the analysis in **Figure 3**, there was a notable rise in diabetes rates as TyG index quartiles increased (P < 0.001). In **Figure 4**, the Kaplan-Meier curves demonstrate the probability of developing diabetes based on the TyG index. Transitioning probabilities varied significantly according to TyG index (p<0.001), with a consistent increase in likelihood as the TyG index rose. This suggests that non-obese young individuals with the highest ratio had a higher chance of developing diabetes.

### Factors influencing risk of diabetes analyzed by univariate cox proportional hazards regression

The univariate analysis showed that the risk of diabetes was positively associated with DBP, age, BMI, AST, SBP, TG, FPG at baseline, ALT, TyG index, and TC, all with a significance level of P<0.05. Conversely, it was negatively associated with HDL-c (all P<0.05; in **Table 2**).

**Table 2:** Risk of diabetes analyzed by univariate Cox proportional hazards regression.

Variable	Characteristics	HR (95% CI)	P-value
Age (years)	35.73 ± 6.38	1.09 (1.08, 1.11)	<0.0001
Gender			
Male	56,469 (45.89%)	Ref	
Female	66,589 (54.11%)	0.51 (0.43, 0.61)	<0.0001
BMI (kg/m2)	21.34 ± 2.09	1.44 (1.37, 1.51)	<0.0001
SBP (mmHg)	113.29 ± 13.47	1.03 (1.03, 1.04)	<0.0001
DBP (mmHg)	70.81 ± 9.48	1.05 (1.04, 1.06)	<0.0001
FPG (mg/dL)	86.79 ± 9.72	1.17 (1.16, 1.18)	<0.0001
TG (mg/dL)	91.36 ± 62.81	1.00 (1.00, 1.00)	<0.0001
TyG index	8.13 ± 0.55	4.58 (4.07, 5.15)	<0.0001
ALT (U/L)	15.00 (11.40-22.00)	1.01 (1.00, 1.01)	<0.0001
AST (U/L)	20.00 (17.00-23.90)	1.01 (1.00, 1.01)	<0.0001
HDL-c (mg/dL)	41.38 (0.00-55.68)	0.996 (0.993, 0.999)	0.0127
TC (mg/dL)	174.93 ± 31.88	1.01 (1.01, 1.01)	<0.0001

HR, Hazard ratios; CI, confidence, Ref, reference.

### The results of multivariable analyses using cox proportional-hazards regression models

Three models were developed using Cox proportional hazards regression to analyze the correlation between the TyG index and the risk of developing diabetes. The initial model, without adjustments, revealed that for every 1-unit rise in the TyG index, there was a 358% increase in the likelihood of progressing to a diabetic state, with a HR of 4.58 (95% CI 4.07-5.15, P<0.0001). In the partially adjusted model, which only considered age and gender, each 1-unit increase in the TyG index showed a 263% rise in the likelihood of developing diabetes, with a HR of 3.63 (95% CI 3.16-4.17, P<0.0001). The fully adjusted model demonstrated that a 1-unit increase in the TyG index was linked to a 257% increase in the likelihood of diabetes, with a HR of 3.57 (95% CI 2.92-4.36, P<0.0001). The confidence intervals’ distribution indicates the robustness of the connection between the TyG index and diabetes risk (**Table 3**). Moreover, we transformed the TyG index from continuous to categorical and incorporated the grouped TyG index back into the analysis. The outcomes from the adjusted multivariate model displayed that in comparison to those in Q1, the HR for individuals in Q2-Q4 were 1.15, 1.63, and 5.5 respectively. This indicates that relative to those in Q1, the risk of progressing to diabetes increased by 15% for Q2, 63% for Q3, and 450% for Q4 participants (**Table 3**, Model II). Upon segregating the total population into Chinese and Japanese subgroups (**Supplementary Tables 3**, **4**), the findings were consistent for both Chinese and Japanese cohorts compared to the overall sample.

**Table 3:** Relationship between TyG index and risk of diabetes in different models.

Exposure	Crude model (HR,95%CI) P	Model I (HR,95%CI) P	Model II (HR,95%CI) P	Model III (HR,95%CI) P
TyG index	4.58 (4.07, 5.15) <0.0001	3.63 (3.16, 4.17) <0.0001	3.57 (2.92, 4.36) <0.0001	3.63 (2.73, 4.83) <0.0001
(quartile)				
Q1	Ref	Ref	Ref	Ref
Q2	1.40 (0.95, 2.06) 0.0870	1.24 (0.84, 1.83) 0.2729	1.15 (0.67, 1.96) 0.6204	1.13 (0.48, 2.69) 0.7775
Q3	2.15 (1.50, 3.07) <0.0001	1.68 (1.16, 2.42) 0.0055	1.63 (0.99, 2.68) 0.0550	1.96 (0.89, 4.32) 0.0964
Q4	9.60 (7.11, 12.97) <0.0001	6.08 (4.39, 8.43) <0.0001	5.50 (3.48, 8.68) <0.0001	4.88 (2.24, 10.64) <0.0001
P for trend	<0.0001	<0.0001	<0.0001	<0.0001

Crude model: we did not adjust other covariates.

Model I: we adjusted age, gender.

Model II: we adjusted age, gender, SBP, DBP, BMI, ALT, AST, TC, HDL-c.

Model III: we adjusted age(smooth), gender, SBP (smooth), DBP (smooth), BMI (smooth), ALT (smooth), AST (smooth), TC (smooth), HDL-c(smooth). HR, Hazard ratios; CI, confidence, Ref, reference.

### Sensitivity analysis

To ensure the reliability of our conclusions, a series of sensitivity analyses were carried out. Initially, Model III of the generalized additive models (GAM) was used, which included additional smoothing terms for different variables and showed a HR of 3.63 (2.73-4.83, P < 0.0001) (**Table 3**, Model III). Following this, individuals with SBP>140 mmHg (3,631 participants) were excluded. After adjusting for confounding variables, the results consistently indicated a positive correlation between the TyG index and the risk of diabetes (HR = 3.62, 95% CI: 2.93-4.47, p < 0.0001). In a subsequent sensitivity analysis, participants with DBP>90 mmHg (N= 3,779) were removed. Even after accounting for confounding factors, the outcomes continued to show a sustained positive relationship between the TyG index and the risk of diabetes (HR=3.63 95% CI: 2.94-4.48, p < 0.0001). An additional analysis focusing solely on male participants revealed a HR of 2.96 (95% CI: 2.33-3.77, p < 0.0001). Our comprehensive sensitivity analyses support the credibility of our findings (**Table 4**).

**Table 4:** Relationship between TyG index and the risk of diabetes in different sensitivity analyses.

Exposure	Crude model I (HR,95%CI) P	Model II(HR,95%CI) P	Model III(HR,95%CI) P
TyG index	3.62 (2.93, 4.47) <0.0001	3.63 (2.94, 4.48) <0.0001	2.96 (2.33, 3.77) <0.0001
(quartile)			
Q1	Ref	Ref	Ref
Q2	1.22 (0.70, 2.12) 0.4824	1.13 (0.65, 1.96) 0.6706	0.46 (0.21, 1.01) 0.0528
Q3	1.68 (1.00, 2.82) 0.0519	1.66 (1.00, 2.77) 0.0505	0.72 (0.37, 1.38) 0.3201
Q4	5.85 (3.64, 9.41) <0.0001	5.52 (3.46, 8.82) <0.0001	2.33 (1.32, 4.11) 0.0035
P for trend	<0.0001	<0.0001	<0.0001

Crude model I was a sensitivity analysis performed after excluding participants with SBP>140 mmHg (N= 3,631). we adjusted age, gender, SBP, DBP, ALT, AST, TC, HDL-c.

Model II was a sensitivity analysis performed after excluding participants with DBP>90 mmHg (N= 3,779). we adjusted age, gender, SBP, DBP, ALT, AST, TC, HDL-c.

Model III was a sensitivity analysis performed on participants without female (N= 66,589). We adjusted age, gender, SBP, DBP, BMI, ALT, AST, TC, HDL-c. HR, Hazard ratios; CI, confidence, Ref, reference.

### Cox proportional hazards regression model with cubic spline functions to account for nonlinearity

**Figure 5:**
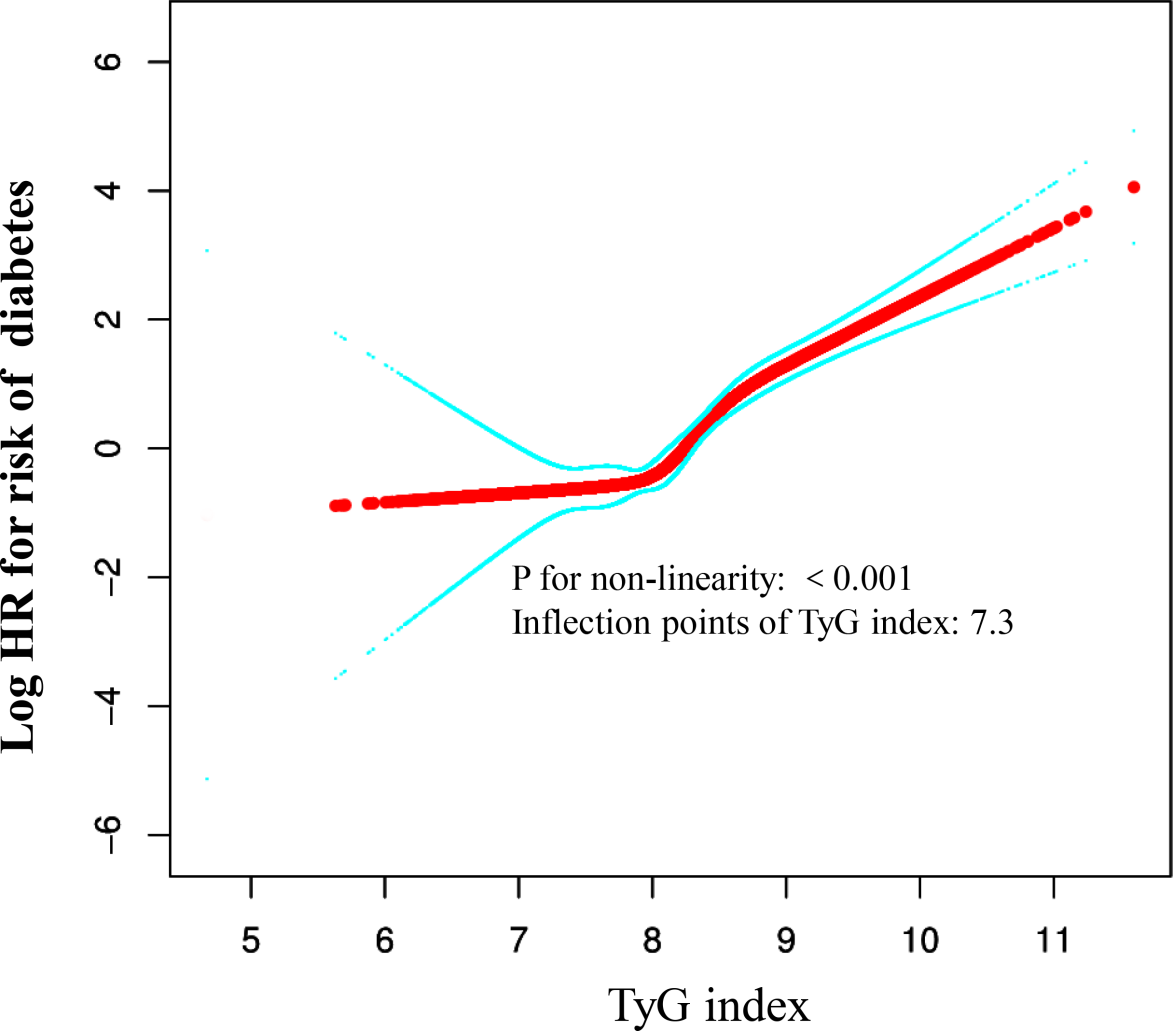
The non-linear relationship between TyG index and the risk of diabetes in non-obese young participants. We used a Cox proportional hazards regression model with cubic spline functions to evaluate the relationship between TyG index and risk of diabetes. The result showed that the relationship between the TyG index and risk of diabetes in non-obese young participants was non-linear, with the inflection point of TyG index being 7.3.

A relationship between the TyG index and the risk of diabetes was observed in our study, as shown in **Figure 5**, **Table 5**. Initially, a Cox proportional hazards regression model was applied using cubic splines to analyze how the TyG index is associated with diabetes risk. The findings pointed towards a nonlinear link between the TyG index and the risk of developing diabetes. To delve deeper into this association, we investigated using a two-piecewise Cox proportional hazards regression model. The standard Cox regression model exhibited a significant HR of 3.57 (95% CI: 2.92-4.36) with a P value of <0.0001, signaling a strong correlation between the TyG index and diabetes risk. Interestingly, a pivotal point at 7.3 for the TyG index was identified. Below this threshold (<6.3), the HR sharply increased to 0.34 (95% CI: 0.06-1.88) with a P value of <0.0001. Conversely, for values equal to or greater than the turning point (≥7.3), the HR was 3.70 (95% CI: 3.02-4.52) with a P value of 0.037.

**Table 5:** The result of the two-piecewise Cox proportional hazards regression model.

Outcome: diabetes	HR, 95%CI P	value
Standard Cox regression	3.57 (2.92, 4.36)	<0.0001
Fitting model by two-piecewise Cox regression		
Inflection points of TyG index	7.3	
<7.3	0.34 (0.06, 1.88)	0.2161
≥7.3	3.70 (3.02, 4.52)	<0.0001
P for trend		0.037

### Subgroup analysis

**Figure 6:**
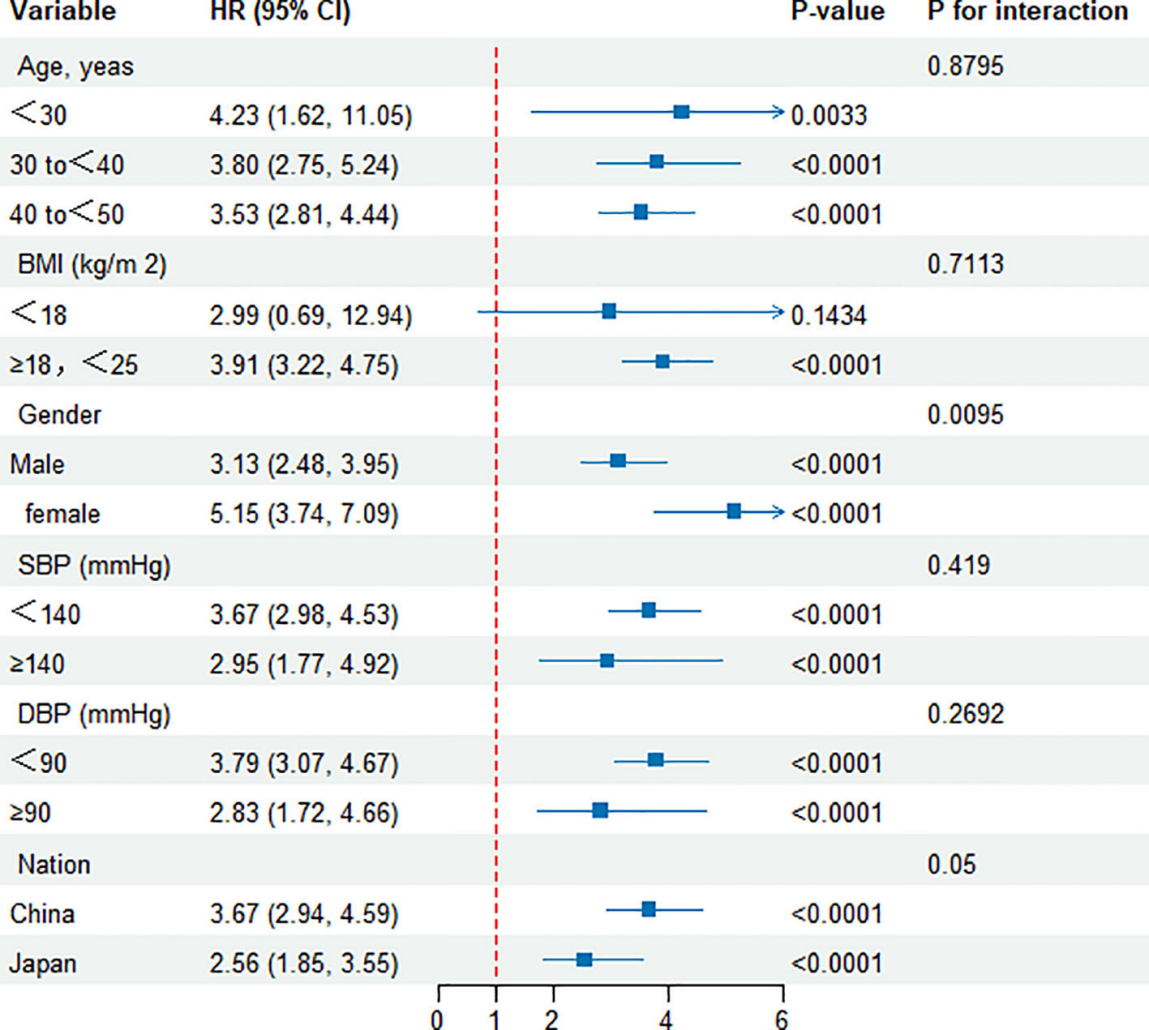
Effect size of TyG index on diabetes in prespecified and exploratory subgroups.

**Figure 6** illustrates a comprehensive subgroup analysis that was performed. Variables such as age, BMI, gender, systolic and diastolic blood pressures, as well as nationality, did not modify the correlation between the TyG index and the likelihood of developing diabetes. Consequently, no substantial correlations were observed between these factors and the TyG index (all interaction P values > 0.05). Furthermore, a subgroup analysis was carried out based on nationality, revealing consistent outcomes for both Chinese and Japanese cohorts.

## Discussion

This retrospective cohort study investigated the association between the TyG index and the risk of diabetes among non-obese young adults. By conducting a long-term follow-up study of 123,058 non-obese young participants, including 113,509 Chinese and 9,459 Japanese, we found a significant increase in risk of diabetes associated with higher TyG index values. Moreover, a L-shaped curve was observed. When the TyG index reached or exceeded 7.3, each additional unit increase in the TyG index was associated with a 270% increased risk of diabetes among non-obese young adults (HR 3.7, 95% CI 3.02-4.52, P < 0.001); whereas for TyG indices below 7.3, an increase in TyG index did not increase the prevalence of diabetes (HR 0.34, 95% CI 0.06-1.88, P < 0.001). This suggests that maintaining a TyG index below 7.3 could potentially reduce the risk of diabetes in non-obese young people.

According to the International Diabetes Federation (IDF), the global prevalence of diabetes is on the rise (29). It is estimated that approximately 8.8% of the global adult population had diabetes in 2013, with the prevalence expected to rise to 10.1% by 2030 (30). In some countries, especially in developed regions, the prevalence of diabetes is particularly high (31). Over the past 20 years, the prevalence of diabetes in China has shown a significant upward trend, from 2.5% in 1994 to 11.6% in 2010, more than doubling (32). Predictions suggest that by 2030, the prevalence of diabetes in China will further increase, potentially affecting up to 42.3 million individuals (33).

Recent years have seen a trend towards younger ages at diabetes onset (34). A study reported that 26.9% of participants under the age of 45 were diagnosed with diabetes or likely diabetes (35). Although obesity is a significant risk factor for diabetes, the incidence of diabetes among the non-obese population is also rising. Research indicates that even individuals who are normal weight or underweight can develop type 2 diabetes (34). It has been observed that while most cases of type 2 diabetes (T2D) occur in obese individuals, a small percentage of T2D patients are underweight or of normal weight (36). Thus, it is crucial for non-obese young adults to identify early signs of diabetes.

Previous studies have commonly utilized the TyG index for the evaluation of insulin resistance (IR) (37). Research results indicate a positive association between the TyG index and diabetes risk within the overall population (38). A cohort study with 201,298 Chinese participants and an average follow-up of 3.12 years revealed that adjusting for relevant factors, Cox proportional hazards regression analysis indicated a 234% higher risk of diabetes for each increment in the TyG index (HR = 3.34, 95% CI 3.11–3.60). Additionally, a notable non-linear association was observed between the TyG index and the likelihood of developing diabetes in the future (20). Another retrospective cohort study involving 25,159 American participants observed that each unit increase in the TyG index increased the risk of diabetes by 181% (HR = 2.81, 95% CI 2.62–3.01), with the association between TyG and the prevalence of prediabetes and diabetes being nonlinear, and the threshold identified at 8.0 (21). Additionally, one study suggested a U-shaped relationship between the TyG index and risk of diabetes in a normoglycemic population, recommending a TyG index of 7.27 for men and 7.97 for women for the lowest risk of diabetes (19). Besides these studies, some have focused on specific populations, including a retrospective cohort study involving 15,464 Japanese participants with different obesity profiles, which, after adjusting for potential confounders, observed that each unit increase in the TyG index was associated with a 70% increased risk of diabetes (HR = 1.70, 95% CI 1.34–2.16) (22). However, to date, no cohort studies have explored the relationship between the TyG index and risk of diabetes among non-obese young adults. Given the large population base in Asian countries, particularly China and Japan, this study aims to explore the association between the TyG index and risk of diabetes among non-obese young adults in these regions.

Our findings align with those of Zhang, et al. and Li, et al. (21, 38). Our study also found a positive correlation between the TyG index and risk of diabetes. In our research, after adjusting for relevant variables, each unit increase in the TyG index was associated with a 257% increase in the risk of diabetes (HR = 3.57, 95% CI 2.92-4.36). However, our study targeted a very large sample of non-obese young individuals in Asia (China and Japan). Furthermore, our study also recorded a nonlinear association and threshold effect between the TyG index and risk of diabetes, but our results showed an L-shaped relationship, with our threshold identified at 7.3. When the TyG index was ≥7.3, the risk of diabetes increased with an increase in the TyG index; whereas when the TyG index was <7.3, an increase in the TyG index did not increase the prevalence of diabetes. This suggests that maintaining a TyG index below 7.3 might reduce the risk of diabetes. When the TyG index is above 7.3, the speed of increase in risk of diabetes accelerates with an increase in the TyG index. Additionally, unlike other studies, our threshold is lower, indicating that early intervention to reduce the TyG index is particularly necessary for the non-obese young adult population to lower the incidence of diabetes.

Our study has significant clinical implications. We have discovered a non-linear association between the TyG index and diabetes risk in non-obese young adults. Monitoring the TyG index can help prevent diabetes. Specifically, when the TyG index reaches or exceeds 7.3, a significant reduction in diabetes risk can be achieved by lowering fasting blood glucose and triglycerides. Conversely, when the TyG index is below 7.3, the incidence of diabetes remains relatively low. Therefore, we recommend early lifestyle modifications for non-obese young adults, including reducing the intake of high-fat foods, increasing physical activity, and managing blood glucose levels to maintain a low TyG index, particularly below 7.3. These measures effectively reduce the risk of developing diabetes.

The substantial difference in HR on either side of this threshold is attributed to physiological and metabolic variations linked to the TyG index. TyG levels above 7.3 may suggest insulin resistance and dyslipidemia, both recognized as precursors to diabetes. Below this threshold, individuals may show enhanced insulin sensitivity and lipid profiles, thereby reducing the risk of diabetes development. This discovery emphasizes the critical need to account for TyG index thresholds when evaluating diabetes risk in non-obese groups. Further research could clarify the mechanisms behind these connections and guide interventions aimed at reducing the TyG index to mitigate diabetes risk.

This study has several significant strengths that merit recognition. First, our research is the first to explore the association between the TyG index and risk of diabetes among non-obese young adults, especially across multiple Asian countries (China and Japan), with a large sample size that enhances the statistical power of the study, thereby increasing the reliability and stability of the findings. Long-term follow-up helps better observe the causal relationships, as continuous observation of the relationship between exposure and outcomes can more accurately assess the impact of exposure factors on outcomes. Second, the study delves into the nonlinear relationship between the TyG index and risk of diabetes and identifies the turning point. Third, a range of extensive sensitivity analyses were carried out to further confirm the results of the research. These analyses included integrating continuous covariates into curves using generalized additive models (GAM). Furthermore, analyses were performed on subgroups and interactions to enhance the validity and consistency of our findings.

However, some potential limitations of the study need to be considered. First, as this is a retrospective study, researchers could not control the data collection and recording process, which may lead to incomplete and inaccurate information retrieval; therefore, conducting randomized controlled trials or prospective studies is imperative. Additionally, retrospective studies are susceptible to selection bias and information bias. Second, the study population included only Asian participants, primarily from China and Japan, which may limit the applicability of the study findings in other regions. Therefore, further research is needed to explore the association between the TyG index and risk of diabetes in different regions, such as the Middle East and India. Third, as this study relied on secondary analysis of published data, it may be affected by issues with the quality of the original data, such as missing, erroneous, or inconsistent data, which could affect the accuracy and reliability of the analyses. And the original data did not include factors such as genetics, lifestyle, and environment, which precluded an assessment of how these variables might influence the association between the TyG index and diabetes risk. Additionally, the researchers could not control for variables in data collection, which may include potential confounders or unconsidered factors that could affect the accuracy of the analysis results. Therefore, these shortcomings need to be cautiously considered and supplemented with other research methods for a comprehensive analysis. Finally, as an observational study, this research established a conjectural association between the TyG index and the risk of diabetes, rather than establishing causality. Care should be taken in interpreting the results, as causality cannot be concluded based solely on this study.

## Conclusion

In the non-obese young adult populations of Asia (China and Japan), this study found a positive correlation and a nonlinear relationship between the TyG index and the risk of diabetes. Specifically, when the TyG index exceeded 7.3, there was a clear positive correlation between the TyG index and risk of diabetes. However, when the TyG index was below 7.3, no trend was observed indicating an increase in risk of diabetes with an increase in the TyG index. Therefore, for non-obese young adults, it is advisable to implement interventions to lower triglycerides or fasting blood glucose levels, effectively reducing the likelihood of a TyG index below 7.3. These intervention measures are expected to significantly reduce the risk of diabetes.

## Data availability statement

The datasets presented in this study can be found in online repositories. The names of the repository/repositories and accession number(s) can be found in the article/**Supplementary Material**.

## Ethics statement

The studies involving humans were approved by the Rich Healthcare Group Review Board. The studies were conducted in accordance with the local legislation and institutional requirements. Written informed consent for participation in this study was provided by the participants’ legal guardians/next of kin.

## Author contributions

JH: Writing – review & editing, Writing – original draft, Formal analysis, Data curation. WD: Writing – review & editing, Formal analysis, Data curation. LC: Writing – original draft, Formal analysis, Data curation. ZH: Writing – review & editing, Formal analysis. CL: Writing – review & editing, Funding acquisition. KW: Writing – review & editing, Formal analysis, Data curation.
