# Supplementary material for: Elevated triglyceride-glucose index associated with increased risk of diabetes in non-obese young adults: a longitudinal retrospective cohort study from multiple Asian countries
Source: Front Endocrinol (Lausanne). 2024 Aug 8;15:1427207. doi: 10.3389/fendo.2024.1427207 (PMC11338785; doi:10.3389/fendo.2024.1427207)
Supplement: Supplementary file 1 [file Table_1.docx]

Supplementary Table 1 The baseline characteristics of participants in Chinese.

| TyG index (quartile) | Q1 (≤7.78) | Q2 (7.79-8.10) | Q3 (8.11-8.47) | Q4 (≥8.48) | P-value |
| --- | --- | --- | --- | --- | --- |
| participants | 28,346 | 28,407 | 28,331 | 28,425 |  |
| Age (years) | 34.36 ± 6.10 | 34.92 ± 6.26 | 35.45 ± 6.34 | 36.97 ± 6.36 | <0.001 |
| BMI (kg/m2) | 20.57 ± 1.97 | 20.98 ± 2.04 | 21.48 ± 2.03 | 22.46 ± 1.80 | <0.001 |
| SBP (mmHg) | 110.16 ± 12.67 | 112.04 ± 13.02 | 114.03 ± 13.29 | 117.83 ± 13.69 | <0.001 |
| DBP (mmHg) | 68.75 ± 8.90 | 69.88 ± 9.10 | 71.25 ± 9.20 | 74.03 ± 9.77 | <0.001 |
| FBG (mg/dL) | 82.38 ± 9.29 | 85.13 ± 9.33 | 87.53 ± 9.01 | 90.51 ± 9.69 | <0.001 |
| TyG index | 7.52 ± 0.21 | 7.95 ± 0.09 | 8.28 ± 0.11 | 8.86 ± 0.35 | <0.001 |
| TG (mg/dL) | 46.12 ± 9.50 | 67.41 ± 9.68 | 91.50 ± 13.37 | 167.94 ± 85.08 | <0.001 |
| ALT (U/L) | 19.60 (17.00-22.70) | 19.90 (17.00-23.00) | 20.45 (17.70-24.00) | 22.20 (19.00-27.00) | <0.001 |
| AST (U/L) | 13.00 (10.20-17.00) | 14.00 (10.80-19.00) | 15.50 (11.70-22.00) | 20.70 (14.40-30.70) | <0.001 |
| TC (mg/dL) | 161.86 ± 27.53 | 169.23 ± 28.84 | 175.24 ± 30.15 | 187.91 ± 33.36 | <0.001 |
| HDL-c (mg/dL) | 34.03 (0.00-57.62) | 38.28 (0.00-56.07) | 39.06 (0.00-54.14) | 36.74 (0.00-50.66) | <0.001 |
| Gender |  |  |  |  | <0.001 |
| Male | 7,491 (26.43%) | 10,521 (37.04%) | 14,034 (49.54%) | 19,766 (69.54%) |  |
| Female | 20,855 (73.57%) | 17,886 (62.96%) | 14,297 (50.46%) | 8,659 (30.46%) |  |
| Follow-up (year) | 3.25 ± 0.97 | 3.14 ± 0.96 | 3.08 ± 0.92 | 3.04 ± 0.90 | <0.001 |
| Incident of diabetes | 33 (0.12%) | 42 (0.15%) | 59 (0.21%) | 271 (0.95%) | <0.001 |

Continuous variables were summarized as mean (SD) or medians (quartile interval); categorical variables were displayed as percentage (%)

Abbreviations: BMI, body mass index; SBP, systolic blood pressure; DBP; diastolic blood pressure; TG triglyceride; AST aspartate aminotransferase; ALT, alanine aminotransferase; FBG, fasting plasma glucose; TyG index, triglyceride glucose index.
