# Supplementary material for: Elevated triglyceride-glucose index associated with increased risk of diabetes in non-obese young adults: a longitudinal retrospective cohort study from multiple Asian countries
Source: Front Endocrinol (Lausanne). 2024 Aug 8;15:1427207. doi: 10.3389/fendo.2024.1427207 (PMC11338785; doi:10.3389/fendo.2024.1427207)
Supplement: Supplementary file 2 [file Table_2.docx]

Supplementary Table 2 The baseline characteristics of participants in Japanese.

| TyG index (quartile) | Q1 (≤7.45) | Q2 (7.46-7.84) | Q3 (7.85-8.25) | Q4 (≥8.26) | P-value |
| --- | --- | --- | --- | --- | --- |
| participants | 2,383 | 2,391 | 2,387 | 2,388 |  |
| Age (years) | 37.78 ± 5.88 | 39.31 ± 5.70 | 39.75 ± 5.51 | 40.64 ± 5.16 | <0.001 |
| BMI (kg/m2) | 19.95 ± 1.98 | 20.60 ± 2.04 | 21.19 ± 2.03 | 22.21 ± 1.83 | <0.001 |
| SBP (mmHg) | 105.70 ± 11.88 | 108.84 ± 12.37 | 111.51 ± 12.88 | 116.42 ± 13.19 | <0.001 |
| DBP (mmHg) | 65.14 ± 8.32 | 67.37 ± 8.93 | 69.47 ± 9.29 | 73.06 ± 9.50 | <0.001 |
| FBG (mg/dL) | 87.54 ± 6.39 | 90.49 ± 6.56 | 92.49 ± 6.66 | 95.51 ± 6.50 | <0.001 |
| TyG index | 7.10 ± 0.30 | 7.65 ± 0.11 | 8.04 ± 0.12 | 8.67 ± 0.35 | <0.001 |
| TG (mg/dL) | 29.00 ± 7.37 | 47.00 ± 6.03 | 67.62 ± 8.84 | 130.66 ± 61.23 | <0.001 |
| ALT (U/L) | 16.00 (13.00-19.00) | 16.00 (13.00-19.00) | 17.00 (14.00-20.00) | 18.00 (15.00-22.00) | <0.001 |
| AST (U/L) | 13.00 (11.00-17.00) | 15.00 (12.00-20.00) | 17.00 (13.00-23.00) | 21.00 (16.00-29.00) | <0.001 |
| TC (mg/dL) | 177.09 ± 27.87 | 186.80 ± 28.98 | 193.95 ± 29.62 | 206.94 ± 31.52 | <0.001 |
| HDL-c (mg/dL) | 63.30 (54.40-73.30) | 58.00 (50.00-68.00) | 52.30 (44.60-61.10) | 45.20 (38.70-53.50) | <0.001 |
| Gender |  |  |  |  | <0.001 |
| Male | 474 (19.89%) | 907 (37.93%) | 1,349 (56.51%) | 1,927 (80.70%) |  |
| Female | 1,909 (80.11%) | 1,484 (62.07%) | 1,038 (43.49%) | 461 (19.30%) |  |
| Follow-up (year) | 5.85 ± 3.55 | 6.12 ± 3.80 | 6.58 ± 3.92 | 6.83 ± 3.94 | <0.001 |
| Incident of diabetes | 6 (0.25%) | 17 (0.71%) | 16 (0.67%) | 71 (2.97%) | <0.001 |

Continuous variables were summarized as mean (SD) or medians (quartile interval); categorical variables were displayed as percentage (%)

Abbreviations: BMI, body mass index; SBP, systolic blood pressure; DBP; diastolic blood pressure; TG triglyceride; AST aspartate aminotransferase; ALT, alanine aminotransferase; FBG, fasting plasma glucose; TyG index, triglyceride glucose index.
