# Supplementary material for: Elevated triglyceride-glucose index associated with increased risk of diabetes in non-obese young adults: a longitudinal retrospective cohort study from multiple Asian countries
Source: Front Endocrinol (Lausanne). 2024 Aug 8;15:1427207. doi: 10.3389/fendo.2024.1427207 (PMC11338785; doi:10.3389/fendo.2024.1427207)
Supplement: Supplementary file 3 [file Table_3.docx]

Supplementary Table 3 Relationship between TyG index and risk of diabetes in different models in Chinese.

| Exposure | Crude model (HR,95%CI) P | Model I(HR,95%CI) P | Model II(HR,95%CI) P |
| --- | --- | --- | --- |
| TyG index | 4.68 (4.12, 5.32) <0.0001 | 4.04 (3.50, 4.66) <0.0001 | 3.55 (2.80, 4.51) <0.0001 |
| (quartile) |  |  |  |
| Q1 | Ref | Ref | Ref |
| Q2 | 1.41 (0.90, 2.23) 0.1375 | 1.31 (0.83, 2.07) 0.2420 | 1.45 (0.65, 3.22) 0.3625 |
| Q3 | 2.16 (1.41, 3.31) 0.0004 | 1.89 (1.23, 2.91) 0.0037 | 1.87 (0.88, 3.98) 0.1040 |
| Q4 | 10.46 (7.29, 15.02) <0.0001 | 8.03 (5.51, 11.72) <0.0001 | 7.08 (3.56, 14.06) <0.0001 |
| P for trend | <0.0001 | <0.0001 | <0.0001 |

Crude model: we did not adjust other covariates.

Model I: we adjusted age, gender.

Model II: we adjusted age, gender, SBP, DBP, BMI, ALT, AST, TC, HDL-c.
