# Supplementary material for: Elevated triglyceride-glucose index associated with increased risk of diabetes in non-obese young adults: a longitudinal retrospective cohort study from multiple Asian countries
Source: Front Endocrinol (Lausanne). 2024 Aug 8;15:1427207. doi: 10.3389/fendo.2024.1427207 (PMC11338785; doi:10.3389/fendo.2024.1427207)
Supplement: Supplementary file 4 [file Table_4.docx]

Supplementary Table 4 Relationship between TyG index and risk of diabetes in different models in Japanese.

| Exposure | Crude model (HR,95%CI) P | Model I(HR,95%CI) P | Model II(HR,95%CI) P |
| --- | --- | --- | --- |
| TyG index | 3.77 (2.84, 5.00) <0.0001 | 3.51 (2.53, 4.86) <0.0001 | 2.24 (1.47, 3.41) 0.0002 |
| (quartile) |  |  |  |
| Q1 | Ref | Ref | Ref |
| Q2 | 2.48 (0.98, 6.28) 0.0563 | 2.17 (0.85, 5.53) 0.1052 | 1.84 (0.72, 4.71) 0.2065 |
| Q3 | 2.07 (0.81, 5.29) 0.1296 | 1.69 (0.65, 4.41) 0.2834 | 1.16 (0.43, 3.08) 0.7706 |
| Q4 | 8.55 (3.71, 19.70) <0.0001 | 6.36 (2.61, 15.48) <0.0001 | 2.90 (1.10, 7.65) 0.0316 |
| P for trend | <0.0001 | <0.0001 | 0.0216 |

Crude model: we did not adjust other covariates.

Model I: we adjusted age, gender.

Model II: we adjusted age, gender, SBP, DBP, BMI, ALT, AST, TC, HDL-c.
